# Histochemical alteration, protein nitration, and antioxidant expression in oysters as early warning indicators for microplastic contamination in the Texas Gulf Coast

**DOI:** 10.1371/journal.pone.0354521

**Published:** 2026-08-12

**Authors:** Rebecca Muñiz, Md Faisal Amin, Md Saydur Rahman

**Affiliations:** 1 School of Earth, Environmental, and Marine Sciences, University of Texas Rio Grande Valley, Brownsville, Texas, United States of America; 2 School of Integrative Biological and Chemical Sciences, University of Texas Rio Grande Valley, Brownsville, Texas, United States of America; Sylhet Agricultural University, BANGLADESH

## Abstract

Microplastics are widely distributed in aquatic environments and have been detected in numerous organisms. Marine bivalves are among the most affected aquatic organisms by microplastics due to their filter-feeding behavior. In this study, 60 oysters were collected from two sites along the Texas Gulf Coast. Fourier transform infrared (FTIR) spectroscopy and Nile red staining were used to detect microplastics in oyster (*Crassostrea virginica*) tissues. We compared field and laboratory samples collected after a two-week depuration period to identify differences in the shape and size of microplastics in oyster tissues. Results from the field sites showed a large number of microplastics, mostly polyethylene (PE) fibers (~3–4 µm in size), in the gills and digestive glands. Conversely, laboratory samples showed a notable decrease in microplastic size (~1–2 µm), reinforcing the argument for cleaner waters. We performed immunohistochemical (IHC) analysis to assess the effects of PE fibers on the expression of 3-nitrotyrosine protein (NTP, a biomarker of protein nitration), superoxide dismutase (SOD), and catalase (CAT) in oyster tissues. IHC results revealed higher expression (*P* < 0.05) of NTP, SOD, and CAT in the tissues of field oysters compared to laboratory oysters, indicating that microplastics induce nitrative stress, which in turn elevates antioxidant enzyme activity in oyster tissues. Using Periodic-acid Schiff staining, we observed less mucus secretion in the tissues of field oysters than in those of laboratory oysters. This occurrence strengthens the argument that microplastics are causing heightened stress and leaving oysters vulnerable in the transport system and in immune function. Biochemical analysis showed significant (*P* < 0.05) differences in the pH of extrapallial (EF) fluid between field and laboratory oysters, but there were no differences in EF glucose levels. Overall, these findings suggest that PE microplastics cause histochemical and biochemical alterations, increased protein nitration, and increased antioxidant expression, which may impair physiological functions in oysters.

## 1. Introduction

Plastic pollution has become a ubiquitous problem across ecosystems, raising concerns around environmental harm and organismal health [1]. Due to the non-biodegradable nature of plastic polymers, degradation results in fragmentation, forming microplastics [2]. In the ocean, microplastic particles were first reported by fishermen in the 1970s; however, the term “microplastics” was not coined until 2002 to describe small (<5 mm), non-biodegradable plastic polymer particles [1]. Microplastics were first detected in marine sediments in the late 1970s on beaches in Canada, Spain, and other countries [2]. Initial studies estimated around 1,000 microplastic pellets per meter of beach, indicating a high level of contamination that has likely increased as plastic production has increased to meet consumer demand [2,3]. Microplastics are considered “sediment-bound pollutants” that affect the bioavailability of heavy metals, thereby exacerbating concerns around pollutant effects [4]. Many factors influence bioavailability, including sediment properties such as grain size and organic matter content; environmental conditions such as pH, temperature, and oxygen levels; the attachment of sediment particles to microplastics; and biological interactions involving organisms such as benthic invertebrates and filter feeders [5–7]. Unfortunately, the bioavailability of organisms can increase as microplastics, and sediments move into the water column. Understanding these processes is essential when examining trophic transfer in aquatic ecosystems [8–10].

Microplastics have become a major threat to marine ecosystems and organisms, causing physical harm to aquatic life and leaching toxic chemicals into the environment [11–15]. Since plastics do not biodegrade and plastic production continues to increase, their widespread presence and accumulation pose a serious threat to water quality and marine life [16–19]. Filter-feeding shellfish are among the most vulnerable to microplastic ingestion because they acquire nutrients by filtering water [20]. Fish ingest food through their digestive tracts or gills, making them susceptible to microplastics [21]. Fish and shellfish are among the most widely consumed seafood worldwide [20,22]. Oysters, specifically, have long been regarded as a culturally important marine species [23–26], dating back to the Republican era in Rome (509 B.C.E. - 27 B.C.E.) and Greece [27]. Although both fish and shellfish can be consumed raw, shellfish are more often consumed this way, raising concerns around microplastic accumulation in their bodies and subsequent transfer to humans [28–31]. In marine organisms, microplastic ingestion can cause various morphological and physiological changes, often leading to stress, and in some cases, death [32,33]. Some research indicates that microplastics can be transmitted from mothers to offspring, impacting reproductive function, including slower sperm motility and fewer larvae [34–36]. Plastics also leach harmful toxins and can increase the concentration of toxic metals, such as mercury, in aquatic organisms [37,38].

In laboratory experiments, microplastic exposure has been shown to disrupt glycolysis pathways, leading to decreased energy reserves [39]. The enzyme glutamate-pyruvate transaminase (GPT), increases under stress, signaling organism distress [40]. Additionally, key lipids such as triglycerides (TG) and cholesterol (CHO) are affected by microplastic exposure [41,42]. As microplastics disturb these lipids, their energy levels decrease [40,43]. Microplastics significantly affect the health of marine organisms, causing numerous harmful effects which have resulted in impacts on human health and economic stability. [44–46]. Microplastics cause physical and internal damage to marine organisms by inducing oxidative and nitrative stress, leading to an imbalance between antioxidants and free radicals within organisms [13,47–50]. This imbalance can harm cells and tissues by damaging proteins, lipids, and DNA, likely disrupting vital metabolic and respiration pathways [40,51].

As microplastics accumulate within organisms, they induce various toxic effects [52]. One major consequence is an increase in reactive oxygen species (ROS), which can overwhelm antioxidant defenses, including enzymes such as superoxide dismutase (SOD), catalase (CAT), and glutathione peroxidase (GPx) [48]. Antioxidants are essential for combating oxidative stress. ROS levels exceeding the body’s capacity to neutralize them result in heightened oxidative stress, tissue damage, impaired metabolism, and reduced physiological functions due to diminished levels of crucial lipids, proteins, and fatty acids [53–56]. Excessive ROS production occurs when microplastics accumulate in the organs, blood, and tissues of marine organisms, leading to protein carbonylation, lipid peroxidation, and cellular or tissue damage [40,56,57]. Specific pathways convert oxygen into ROS, but when microplastics interfere, ROS levels spike significantly. An imbalance between ROS and reactive nitrogen species (RNS) can damage cellular structures, including proteins (protein carbonylation), DNA, and lipids (lipid peroxidation), potentially leading to mutations [40,56,57]. Increased ROS can also cause mitochondrial dysfunction, which hampers energy production and damages mitochondrial DNA, compromising genetic integrity and leading to mutations, abnormal gene expression, and other genetic disorders [58,59]. This effect is particularly harmful to organisms such as fish and shellfish that rely on sensitive oxygen-dependent pathways.

Oxidative stress occurs when ROS and RNS levels exceed the body’s antioxidant defenses [60,61]. Marine organisms combat oxidative stress through key antioxidant enzymes, such as SOD and CAT, which neutralize ROS and prevent damage [60]. These enzymes help maintain redox balance, keeping oxidation and reduction processes in check [62,63]. SOD converts the toxic superoxide anion (O_2_⁻) into hydrogen peroxide (H_2_O_2_). This step is crucial because the superoxide anion is highly reactive and can damage cellular components such as proteins, lipids, and DNA. By preventing the buildup of free radicals, SOD helps reduce oxidative stress. Then CAT breaks down H_2_O_2_ into water and oxygen, preventing further oxidative damage. This process is important because excess H_2_O_2_ can form harmful hydroxyl radicals (OH·). The ability of CAT to decompose H_2_O_2_ is essential for maintaining redox balance and preventing oxidative stress caused by excessive ROS [64–67]. Under oxidative stress, the production of antioxidant enzymes is often impaired, impacting reproduction, health, and survival [56,68].

Several factors influence enzymes’ function under oxidative stress induced by microplastics, including exposure duration and polymer composition. The longer an organism is exposed to microplastics, the higher the levels of oxidative stress markers increase, resulting in prolonged activity of CAT and SOD [53,69]. This prolonged exposure can be harmful, as the organism’s antioxidant defenses may become overwhelmed, thereby reducing enzyme effectiveness [70,71]. The type and composition of microplastics determine whether antioxidant enzymes are upregulated or downregulated, suggesting the organism’s response varies by composition and environmental context [72,73]. Many studies have examined oxidative and nitrative stress, as well as antioxidant enzyme activity, in various aquatic organisms. The recovery of organisms after exposure to microplastics has also been studied. For instance, a study showed that Nile tilapia (*Oreochromis niloticus*) exposed to different concentrations of microplastics (1, 10, and 100 mg/L) for 15 days had increased SOD and CAT levels in their tissues, indicating oxidative stress [53]. Following exposure, a 15-day recovery period without microplastics (i.e., depuration) was conducted. Blood samples during the depuration phase showed that SOD and CAT levels returned to normal in the tilapia. This result demonstrates that recovery is possible if exposure is reduced or halted. Moreover, depuration can mitigate microplastic-induced oxidative stress in fish [53], suggesting potential resilience among aquatic organisms under suitable environmental conditions.

Eastern oysters (*Crassostrea virginica)* are a commercially and ecologically important species worldwide, particularly along the Texas Gulf Coast [31]. Aside from their economic value, oysters filter large quantities of water, with individual oysters capable of filtering up to 50 gallons per day, thereby improving water quality and reducing turbidity [74]. Additionally, oysters form foundational structures that support the colonization of other organisms and promote sedimentation. Along the Texas Gulf Coast, oyster populations have faced significant decline due to factors including overharvesting, storm events, parasites and viruses, ocean acidification, and anthropogenic activities such as dredging and boating [31]. Plastic debris has also emerged as a critical concern, especially in areas where accumulation rates are higher, like the Texas coast [31]. Given their filter-feeding behavior and ecological importance, oysters are particularly vulnerable to microplastic exposure and serve as valuable bioindicators of plastic pollution in coastal ecosystems.

Although many studies have investigated how microplastics influence oxidative stress and antioxidant responses in aquatic animals, most do not clearly explain what these biomarkers mean in biological terms [58,75,76]. For example, while increases in SOD and CAT are commonly observed, there is often little discussion about what these changes imply for the organism’s overall health. While oxidative and nitrative stress are known to lead to toxic buildup and damage to cells and tissues, the long-term consequences for the organisms remain unclear. The aims of our study were three-fold. First, we examined the presence of microplastics in the tissues of wild-caught Eastern oysters (*Crassostrea virginica)* collected from the Texas Gulf Coast. Second, we assessed the recovery and persistence of microplastics in oyster tissues under controlled laboratory conditions through a depuration study. Third, we evaluated the effects of microplastics on nitrative stress and antioxidant status in oyster tissues. The results of this study provide crucial information on microplastic pollution in wild oyster populations and offer a novel perspective on microplastic recovery and persistence in oysters, helping to inform managers and restoration efforts.

## 2. Materials and methods

### 2.1. Ethical statement

All experimental oysters were collected from the Texas Gulf Coast according to the approved protocol by the Texas Parks & Wildlife Department and cared for in a laboratory experiment according to the guide for Care and Use of Laboratory Animals in the United States National Research Council Committee (https://grants.nih.gov/grants/olaw/guide-for-the-care-and-use-of-laboratory-animals.pdf). The UTRGV Institutional Animal Care and Use Committee (IACUC) does not require any animal care and research protocol for aquatic invertebrates, including oysters.

### 2.2. Experimental animals and sampling sites

Two batches (field and laboratory depuration studies) of oysters (30 oysters per site) were collected from the Texas Gulf Coast: South Padre Island (SPI: 26°4’33” N, 97°9’57” W) and San Martin Lake (SML: 26°0’7” N, 97°180’0” W) in Brownsville, Texas (Fig 1A). The sampling site of SPI was located on the western coast of the Gulf of Mexico, near a firework display platform and largely attributable to human activities such as recreational and commercial fishing. The SPI sampling site contains substantial plastic debris (Fig 1B), which has been documented to split into small plastic pellets (i.e., nurdles) along the shoreline throughout the Gulf of Mexico [77,78]. SML is a shallow-water estuary influenced by tides from the Gulf of Mexico and by wastewater runoff from local farms and the Brownsville wastewater treatment plants [79]. The SML system constitutes a unique environment for microplastics research, as it contains numerous plastic debris items from human activities such as recreational fishing (Fig 1C).

**Fig 1:**
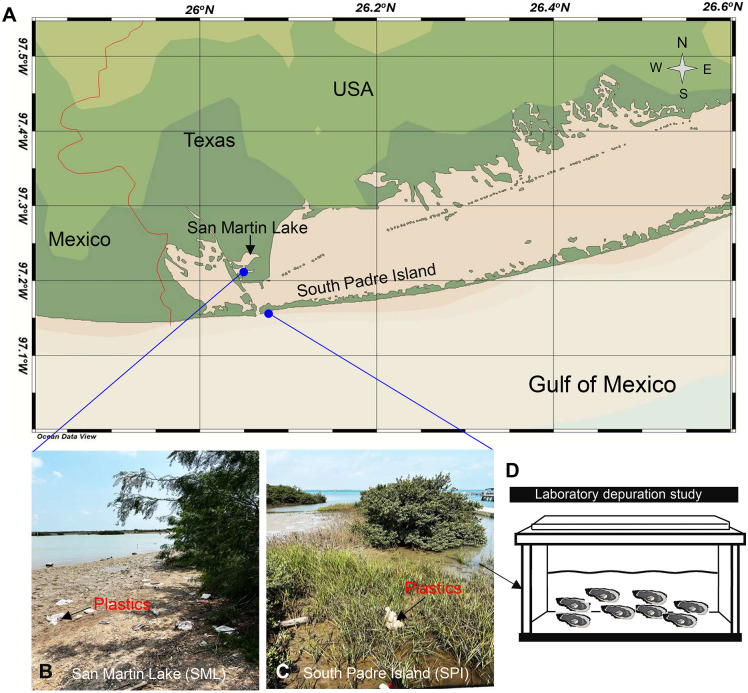
Oyster collection sites on the Texas Gulf Coast. (A) Gulf of Mexico, a marginal sea basin largely surrounded by the North American continent. (A) Blue dots indicate sampling sites. (B, C) Plastic debris of various sizes in sampling sites at San Martine Lake (SML, location: 26°00′10″N, 97°17′56″W) and South Padre Island (SPI, location: 26°04′30″N, 97°09′59″W). (D) Laboratory depuration study for two weeks. Oysters were collected from SPI and SML and kept in filtered seawater in aquariums for two weeks.

### 2.3. Field study: Tissue collection and preservation

The first batch of oysters was collected and brought back to the University of Texas Rio Grande Valley (UTRGV) wet laboratory, where they were immediately sacrificed (SPI: length: 7.43 ± 1.3 cm, width: 3.69 ± 0.48 cm; SML: length: 8.23 ± 1.19 cm, width: 4.54 ± 0.65 cm). The oysters were handled carefully, quickly chilled in ice water to minimize sensation and humanely sacrificed as their shells were shucked with a specialized knife, and their length and width were recorded before they were discarded. Their digestive glands and gills were collected using small blades, scalpels, and tweezers, and their extrapallial (EP) fluid was obtained with a sterilized pipette and stored in 1.5 mL tubes. After collection, tissue samples were preserved in histology cassettes for further analysis. Multiple samples of digestive gland and gill tissues were collected from each oyster and placed in separate dry ice boxes. During collection, the test tubes were kept in dry ice and stored at −80°C, while the cassettes were stored in 4% paraformaldehyde solution (Acros Organics, New Jersey, USA) at 4°C for one week.

### 2.4. Laboratory depuration study

The second batch of oysters was also collected and transported to the UTRGV wet laboratory, where they were kept separately in glass aquariums for two weeks (SPI: length: 6.87 ± 0.9 cm, width: 3.5 ± 0.39 cm; SML: length: 7.5 ± 1.13 cm, width: 4.3 ± 0.93 cm). Four aquariums housed the oysters: two contained SPI oysters, and the other two contained SML oysters (15 oysters per site for a total of 30) (Fig 1D, 1E). The aquariums, filled with 20 gallons of filtered seawater (i.e., a 600-gallon capacity of a multistage and microfiltration unit to remove external plastic debris and microscopic algal cells from seawater), were provided with continuous aeration using air stones and maintained at a controlled temperature of 22°C and a 12-hour light:12-hour dark cycle. The aquariums were siphoned and cleaned by replacing the old water with fresh, aerated seawater. The oysters were fed with frozen marine cuisine (5% of body weight) every other day (Marine Cuisine, California, USA). Hydrological parameters were measured twice daily (temperature: 22.38 ± 0.04 °C, dissolved oxygen: 7.73 ± 0.11 mg/L, pH: 7.85 ± 0.03; salinity: 34 ppt) using a YSI device, once in the morning and once in the afternoon. After two weeks, the gills, digestive glands, and EP fluid were collected using the same procedures as the field samples.

### 2.5. Tissue preservation and histological analysis

After fixation in 4% paraformaldehyde for 1 week, the tissues were dehydrated through an alcohol and xylene series. The samples were placed in glass vials and soaked in ethanol solutions at 50%, 75%, 95%, and 100% for 30 minutes each. The 100% ethanol step was performed twice. During dehydration, samples were shaken at 180 rpm to ensure thorough penetration. Next, samples were immersed in xylene twice, for 15 minutes each, using fresh xylene each time. Then, half of each vial (including the used xylene) was filled with xylene, the other half with melted paraffin wax, and the vials were left overnight at room temperature. The following day, xylene was replaced with fresh paraffin wax, which was poured into the vials and left for one hour. This process was repeated three times with fresh wax each time. Tissue blocks were created by embedding the samples in paraffin wax using plastic cassettes, then cooling. Sections 7 µm thick were cut with a rotary microtome, floated on warm water to expand, mounted onto positively charged slides, and dried on a hot plate overnight. The slides were then ready for further analysis.

### 2.6. Tissue digestion, microplastics assimilation, FTIR spectroscopy and SEM analyses

Before tissue digestion and microplastic analysis, all workplace surfaces were cleaned with 70% ethanol, glassware was rinsed with distilled water, and glassware was covered with aluminum foil. To avoid exogenous microplastic contamination, all experiments were also performed in a closed-door laboratory, and cotton clothing was worn throughout the work period. Gill and digestive gland tissue samples (~0.5 g) were dried in an oven at 65 °C for 24 hours, and then 0.05 M FeSO_4_ (20 mL) and 30% H_2_O_2_ (20 mL) were added to a glass beaker and stirred for 1 hour at room temperature. Afterwards, a 300 mL ZnCl_2_ solution (1.5 g/mL) was added to the mixture, stirred for 1 hour, and then allowed to settle overnight to separate microplastics from tissue samples by floatation. The mixture was filtered through a 0.25 µm cellulose filter (Whatman Grade 4; Cytiva, 47 mm diameter, Marlborough, Massachusetts, USA) under vacuum to recover floating plastic particles. The filter paper was inspected under a light microscope, and images were captured with a digital camera attached to the microscope (AmScope, Irvine, California, USA). The polymer composition was characterized using an attenuated total reflectance Fourier transform infrared (ART-FTIR) spectroscopy (Vertex 70, Bruker Optik GmbH, Ettlingen, Germany). The spectra were acquired from 400 to 4500 cm^-1^ at a resolution of 4 cm^-1^. The spectra were analyzed using Open Specy, an extensive library containing 40,000 open-source Raman and FTIR spectra [80], and were matched against the polymer [81]. A scanning electron microscope (SEM) (SNE-4500M Plus, Southwest Systems, Inc., Anna, Texas, USA) was used to observe the surface texture of microplastics.

### 2.7. Nile Red staining to detect microplastics in oyster tissues

Nile Red, a fluorescent dye for microplastics analysis [82], was prepared at 1 mg/mL in acetone, with a working solution of 5 µg/mL. The stock solution was made by dissolving 0.001 g of Nile red in 1000 µL of acetone. The stock solution was mixed with 250 µL of water and 750 µL of glycerol to make 1 mL of working solution. All procedures were performed in the dark, according to the methods described by de Guzman et al. [83] and Dowarah et al. [84]. After applying the dye to the tissue sections, coverslips were placed on top, and the sections were incubated in the dark for 30 minutes. Fluorescence images were captured with a Zeiss Axioscope microscope (Carl Zeiss Microscope, LLC, White Plains, New York, USA), with settings optimized for microplastic detection using Nile Red. Typically, 5–10 images were taken of each slide, including both gills and digestive gland tissues from field and laboratory oysters (i.e., depuration study).

### 2.8. Periodic acid-Schiff staining for detection of mucous secretion in oyster tissues

The periodic acid-Schiff (PAS) staining technique was used to detect mucous secretion in gill and digestive gland tissues, following the methods described by Steinke et al. [85] and Bancroft and Gamble [86]. Briefly, tissue sections were first deparaffinized in xylene three times for 5 minutes each, then rehydrated through graded ethanol solutions (100%, 95%, 75%, and 50%) for 5 minutes each, followed by two 10-minute immersions in deionized (DI) water. The slides were oxidized with 0.5% periodic acid for 8 minutes, then rinsed three times in DI water for 3 minutes each. Before adding Schiff’s reagent for 20 minutes, the slides were covered with aluminum foil in the dark to protect the fluorescent dye. They were then washed in tap water for 30 minutes and counterstained with 1% methyl green for 1 minute (not exceeding this time). Finally, the slides were rinsed in tap water for 3 minutes and briefly dipped in DI water. The dehydration process involved washing the slides twice for 5 minutes each in 100% ethanol, followed by three immersions in xylene. A coverslip was placed on each slide, and the slides were then allowed to dry. The prepared slides were examined under a light microscope, and images were captured with a digital camera (AmScope). Mucous secretion was identified in tissue images as magenta-stained areas, generally considered to contain neutral mucopolysaccharides, and was quantified using ImageJ software according to the method described by Schneider et al. [87].

### 2.9. Immunohistochemical analysis for NTP, SOD, and CAT expressions in oyster tissues

Immunohistochemical procedures were used to measure three bioindicators: NTP, SOD, and CAT. Although their expressions varied, the protocol remained consistent, with only minor changes to the antibodies. The tissue slides were deparaffinized by immersing them in xylene three times for 5 minutes each, followed by rehydration through ethanol solutions at 100%, 95%, 75%, and 50%. After cleaning, the slides were washed in 1x phosphate buffered saline (PBS) three times for 15 minutes each. A 1% bovine serum albumin (BSA) solution was applied to the slides, which were then incubated for 1 hour at room temperature. Afterwards, the BSA was rinsed off, and the slides were washed in 1x PBS for 5 minutes. The primary antibody (dilution 1:100), specific for NTP, SOD, or CAT (mouse anti-NTP, Santa Cruz Biotechnology, Dallas, TX, USA; rabbit anti-SOD, or rabbit anti-CAT, Novus Biologicals, Centennial, CO, USA), was applied to slides and incubated at 4°C for 48 hours. Following incubation, the slides were washed in 1x PBS three times for 15 minutes each, then secondary antibody (dilution 1:100; NTP: anti-mouse, SOD: anti-rabbit, CAT: anti-rabbit; Cell Signaling, Denver, MA, USA) was applied for 2 hours at room temperature. The slides were then washed again in 1x PBS three times for 15 minutes each, and 3,3-diaminobenzidine (DAB) substrate was added for approximately 3 minutes in the dark. The DAB substrate enabled the detection of NTP, SOD, and CAT expressions in tissues. The slides were washed in DI water for 5 minutes, dehydrated through ethanol solutions at 50%, 75%, 95%, and 100% for 10 minutes each, and then submerged in xylene three times for 10 minutes each. Glass coverslips were placed at the end, and the slides were examined under a light microscope, with images captured using an attached digital camera (AmScope, Irvine, California, USA). The immunostaining (IS) signal was quantified using optical density (OD) measurements in ImageJ, as described by Schneider et al. [87].

### 2.10. EP fluid pH and glucose levels

The pH of field samples was measured by placing the 1.5 mL tubes filled with EP fluid into the Legend Micro 21R Centrifuge for 5 minutes at 5 rpm, allowing cells to settle at the bottom. The tubes were carefully removed, and the EP fluid (500 mL) was collected with a pipette. The pH was then measured using a sensitive pH meter (Eutech Instruments, Waltham, MA, USA). Glucose levels were assessed in EP fluid (15 µL) using the HemoCue Glucose 201 analyzer, according to manufacturer guidelines (Angel Holm, Sweden).

### 2.11. Statistical analysis

Statistical analysis was performed with GraphPad Prism software (GraphPad Software Inc., San Diego, CA, USA). Before analysis, all data were checked for outliers using the ROUT (robust regression followed by outlier identification) method and for normality using the Shapiro-Wilk test. Outlier data points were excluded to prevent distortion of group differences. To compare groups, one-way analysis of variance (ANOVA) and Tukey’s test were used to identify significant differences (*P* < 0.05) for normally distributed data. For non-normally distributed data, the Kruskal-Wallis’s test with Dunn’s test was used for multiple comparisons. Replicated results were combined for a valid statistical comparison.

## 3. Results

### 3.1. Detection of microplastics in tissues of oysters

Various sizes of microplastics were detected in both gills and digestive glands of wild-caught oysters (Fig 2A, 2B). SEM analysis found varied morphologies, including surface roughness, cracks, and spherical particles in the gills and digestive glands (Fig 2C, 2D). FTIR analysis showed the presence of ingested PE microplastics in tissues (Fig 2E, 2F).

**Fig 2:**
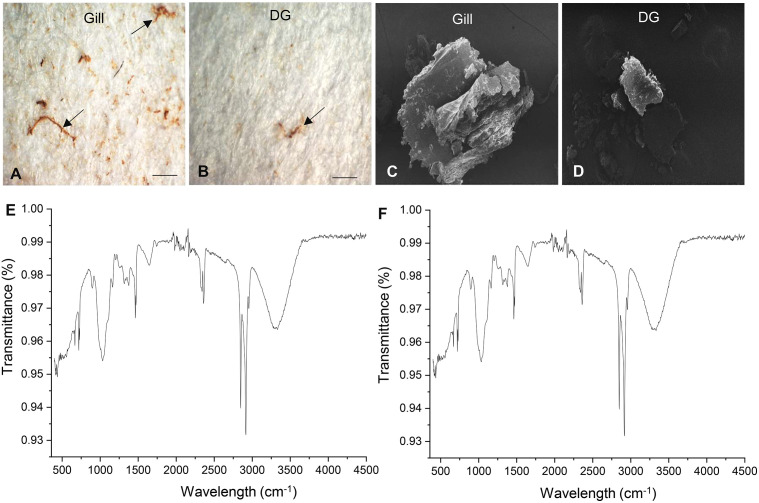
FTIR spectra and SEM images of microplastics in tissues of wild-caught oysters. Photographs of different microplastics in gills (A) and digestive glands (B). Irregular and regular-shaped polyethylene (PE) fragments were detected in gills (C) and digestive glands (D), respectively. FTIR spectra of PE polymer microplastics detected in gills (E) and digestive glands (F). Scale bar = 100 µm.

A significant difference (*P* < 0.05) in microplastic sizes was observed in the gills and digestive glands of oysters from field sites (South Padre Island, SPI; and San Martin Lake, SML) compared to the laboratory depuration study (LDS) (Fig 3). No significant differences in microplastic sizes were found between oysters from SPI and SML in either (Fig 3A-3D, 3I, 3J). Specifically, gill samples from SPI contained significantly larger (*P* < 0.05) microplastics (mean 3.78 ± 1.45 μm) than LDS groups (mean 1.59 ± 0.53 μm), approximately 2.2 times smaller (Fig 3A, 3E, 3I). Similarly, SML gill samples showed significantly larger (*P* < 0.05) microplastics (mean 3.46 ± 1.36 μm) than LDS samples (1.57 ± 0.63 μm), about 2.21 times smaller (Fig 3B, 3F, 3I). Microplastics in the digestive glands of oysters from SPI were also significantly larger (*P* < 0.05) (mean 3.69 ± 1.79 μm) compared to LDS (1.62 ± 0.65 μm), roughly 2.3 times smaller (Fig 3C, 3G, 3J). Likewise, field samples from SML exhibited significantly larger microplastics in the digestive glands (mean 3.32 ± 1.28 μm) than those from LDS (1.6 ± 0.74 μm), approximately 2.1 times less (Fig 3D, 3H, 3J).

**Fig 3:**
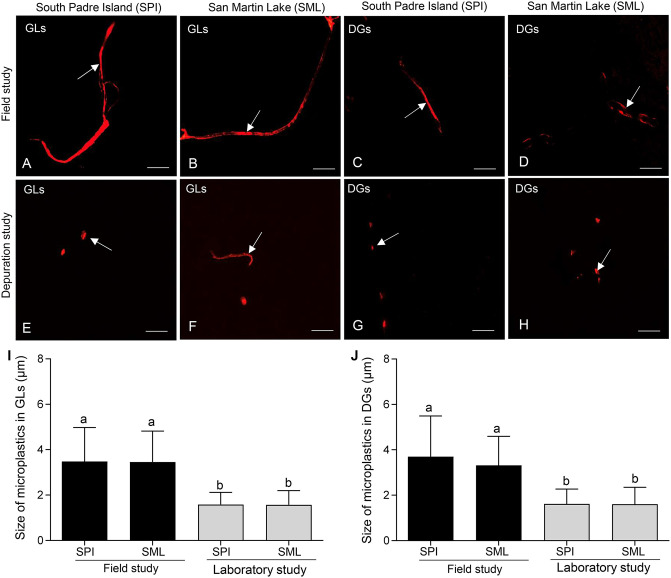
Detection and size of microplastics in tissues of wild-caught oysters collected from field and laboratory studies. Red fluorescent signal and arrows indicate microplastics in the gills (GLs: A, B, E, F) and digestive glands (DGs: C, D, G, H) of oysters collected from South Padre Island (SPI) and San Martin Lake (SML) in the Texas Gulf Coast. A-D: field study, E-H: depuration study. Scale bar = 100 µm. (I, J) Size of microplastics in the gills and digestive glands of oysters. Each value represents the mean ± SD (N = 87-105). Different letters indicate significant differences (Kruskal-Wallis test with Dunn’s multiple comparison test, *P* < 0.05).

### 3.2. Impacts of microplastics on nitrotyrosin protein expression

To evaluate whether microplastics induce nitrative stress, 3-nitrotyrosin protein (NTP) expression was examined in oyster tissues using immunohistochemistry (IHC). The immunostaining (IS) intensity, indicated by brown staining shown by arrows in Fig 4A-4J, demonstrated significant differences (*P* < 0.05) in NTP expression between field sites and the LDS, across gills and digestive glands. In SPI, gill tissues from field samples showed significantly higher (*P* < 0.05) NTP expression (mean OD 0.87 ± 0.13) than LDS tissues (mean OD 0.82 ± 0.16) (Fig 4A, 4C, 4I). Similarly, SML gill tissues had higher NTP expressions (mean OD 0.76 ± 0.18) than LDS samples (mean OD 0.7 ± 0.15) (Fig 4B, 4D, 4I). Similar to gills, digestive glands from SPI also showed significantly higher (*P* < 0.05) NTP expression (mean OD 0.9 ± 0.15) compared to LDS tissues (mean OD 0.8 ± 0.19) (Fig 4E, 4G, 4J). No significant difference was observed in digestive glands between field (SML) and LDS samples (field OD 0.85 ± 0.15, LDS OD 0.98 ± 0.17) (Fig 4F, 4H, 4J). No signals were detected in negative controls (Fig 4K, 4L).

**Fig 4:**
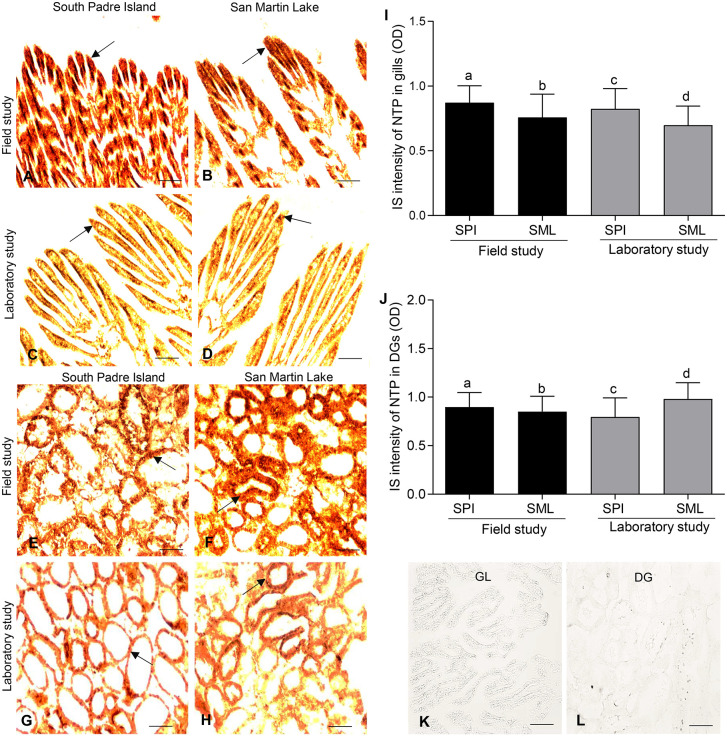
Detection of NTP expression in tissues of wild-caught oysters collected for field and laboratory studies. Arrows indicate NTP expression in the gills (A-D) and digestive glands (E-F) of oysters collected from South Padre Island (SPI) and San Martin Lake (SML) in the Texas Gulf Coast. (I, J) Immunostaining (IS) intensity of NTP in the gills (GLs) and digestive glands (DGs). Each value represents the mean ± SD (N = 184-214). (Kruskal-Wallis test with Dunn’s multiple comparison test, *P* < 0.05). (K, L) Negative control of NTP expression in the gill and digestive gland of oysters. NTP, 3-nitrotyrosin protein; OD, optical density. Scale bar = 100 µm.

### 3.3. Impacts of microplastics on mucus secretion

The impact of microplastics on mucus secretion was assessed by PAS staining of tissues from oysters collected from SPI, SML, and LDS (Fig 5). Significant differences in mucus secretion appeared in the gills of oysters from field sites versus LDS, with oysters from SPI showing less mucus than LDS samples, about 1.6 times less mucus (Fig 5I). Similarly, gill tissues from SML oysters showed significantly lower mucus secretion in field samples (both SPI and SML) compared to LDS, about 1.4 times lower (*P* < 0.05). A significant difference (*P* < 0.05) in mucus secretion was also observed in the digestive glands between field and LDS samples (Fig 5J). In SPI, digestive glands from field samples showed mucus secretion about 5.6 times lower than LDS, which was statistically significant (*P* < 0.05). The SML digestive gland did not show a significant difference but still had slightly less mucus secretion than LDS samples, about 1.1 times lower (Fig 5J).

**Fig 5:**
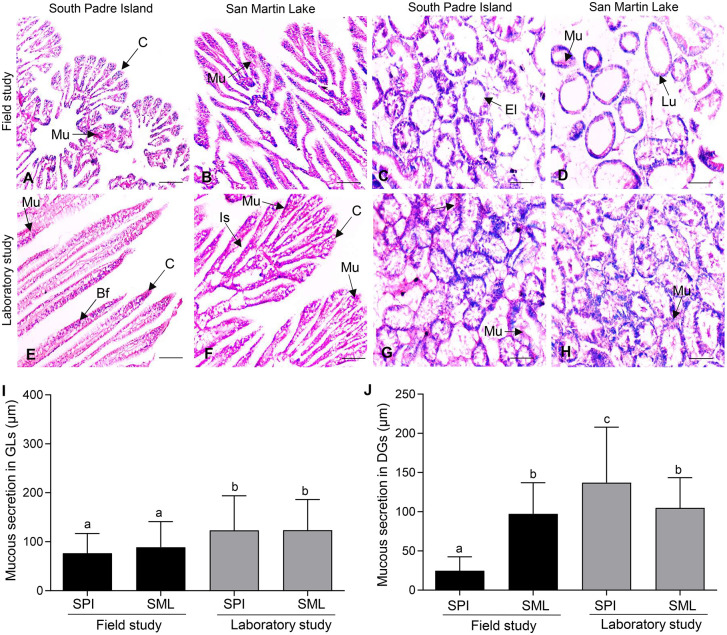
Detection of mucous in tissues of wild-caught oysters collected for field and laboratory studies. Arrows indicate mucous in the gills (A, B, E, F) and digestive glands (C, D, G, H) of oysters collected from South Padre Island (SPI) and San Martin Lake (SML) in the Texas Gulf Coast. Scale bar = 100 µm. (I, J) Area (µm) of mucous in the gills (GLs) and digestive glands (DGs). Each value represents the mean ± SD (N = 52-101). (Kruskal-Wallis test with Dunn’s multiple comparison test, *P* < 0.05). Different letters indicate a significant difference. Bf, branchial filament; C, cilia, Is, intra-lamellar space; El, epithelial lining; Mu, mucus; Lu, lumen.

### 3.4. Impacts of microplastics on body fluid conditions

To evaluate how microplastics affected body fluid conditions in oysters, both pH and glucose levels were measured in extrapallial (EP) fluid (Fig 6). In SPI oysters, EF fluid pH was 7 ± 0.08, significantly higher (*P* < 0.05) than LDS samples, which measured 7.2 ± 0.08. Similarly, the EF fluid pH in SML was 7.14 ± 0.1, compared to 7.12 ± 0.03 in LDS, roughly 1.01 times higher (Fig 6A). Although glucose levels varied slightly, the differences were not statistically significant in either field or laboratory EF fluid samples (Fig 6B).

**Fig 6:**
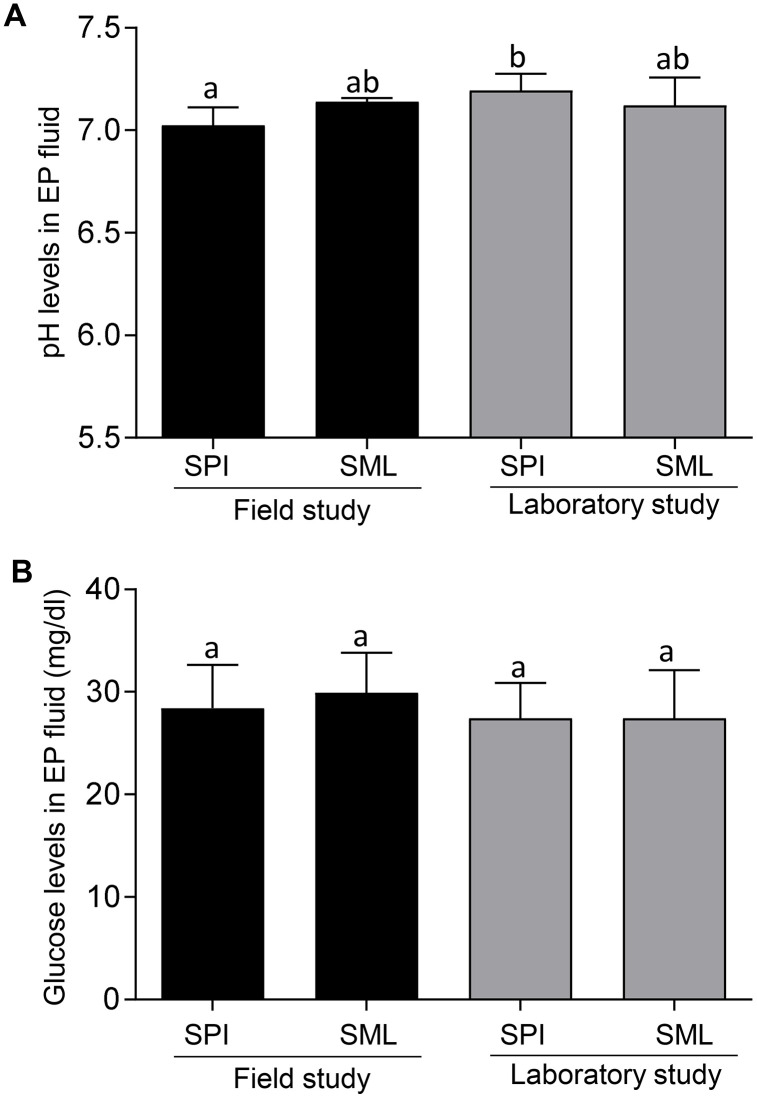
pH and glucose levels in extrapallial fluid of wild-caught oysters collected for field and laboratory studies. (A) pH and (B) glucose levels in extrapallial (EP) fluid of oysters collected from South Padre Island (SPI) and San Martin Lake (SML) in the Texas Gulf Coast. Each value represents the mean ± SD (N = 7–15). Different letters indicate significant differences (one-way ANOVA followed by Tukey’s test, *P* < 0.05).

### 3.5. Impacts of microplastics on antioxidant enzyme expression

To elucidate the potential adverse effects of microplastics, the expression of two key antioxidant enzymes, superoxide dismutase (SOD) and catalase (CAT), was analyzed in the gills and digestive glands of oysters collected from SPI, SML, and LDS (Fig 7). Oysters from both SPI and SML showed higher SOD expression in their gill tissues (Fig 7A, 7B). The IS intensity of SOD expression was significantly higher (*P* < 0.05) in SPI oysters (mean OD 0.79 ± 0.15) than in LDS samples (mean OD 0.65 ± 0.13), about 1.2 times higher (Fig 7A, 7C, 7I). Likewise, gill tissues from SML exhibited higher IS intensity (~1.1 times) than those from LDS samples (field OD: 0.8 ± 0.02, LDS OD: 0.73 ± 0.01) (Fig 7B, 7D, 7I). Similar trends were observed in the digestive glands of SPI (Fig 7E, 7G) and SML (Fig 7F, 7H). SOD expression was significantly higher in field samples than in laboratory ones (SPI – field: 0.92 ± 0.22, laboratory: 0.893 ± 0.19; SML – field: 0.72 ± 0.19, laboratory: 0.75 ± 0.16) (Fig 7J). No IS signals appeared in the negative controls (Fig 7K, 7L).

**Fig 7:**
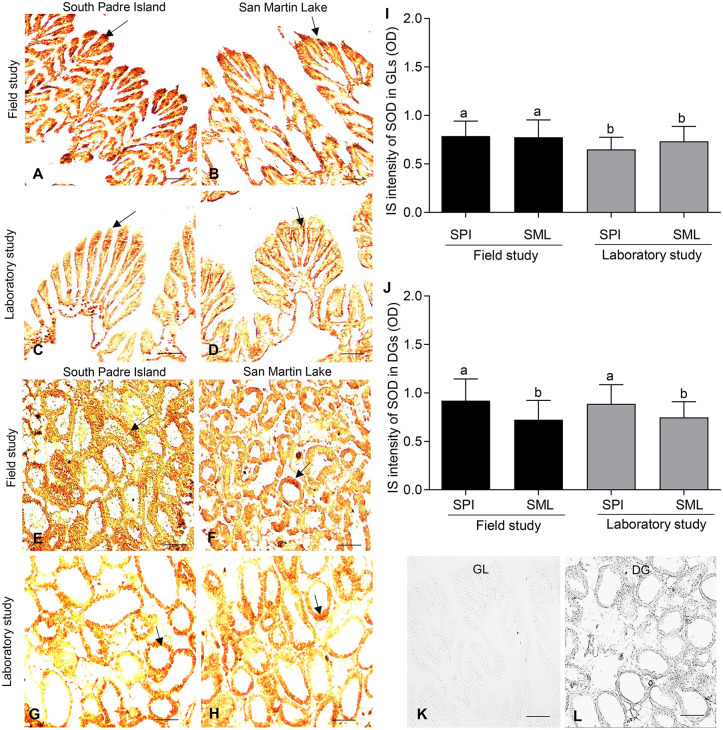
Detection of superoxide dismutase (SOD) in tissues of wild-caught oysters collected for field and laboratory studies. Arrows indicate SOD expression in the gills (A-D) and digestive glands (E-F) of oysters collected from South Padre Island (SMI) and San Martin Lake (SML) in the Texas Gulf Coast. (I, J) Immunostaining (IS) intensity of SOD in the gills (GLs) and digestive glands (DGs). Each value represents the mean ± SD (N = 191-213). Different letters indicate significant differences (Kruskal-Wallis test with Dunn’s multiple comparison test, *P* < 0.05). (K, L) Negative control of SOD expression in the gill (K) and digestive gland (L) of oysters. OD, optical density. Scale bar = 100 µm.

CAT expression in gills and digestive glands was also analyzed, as shown in Fig 8 with arrows indicating areas of dark brown staining that mark expression zones. Field samples from both SPI and SML showed greater CAT expression than LDS samples (Fig 8A-8H). Significant differences in IS intensity of CAT expression were detected across all gill and digestive gland tissues between field and laboratory samples (*P* < 0.05, Fig 8I, 8J). In SPI samples, gill tissues showed higher CAT IS intensity than LDS samples (field OD: 0.95 ± 0.15, LDS OD: 0.72 ± 0.16, about 1.3 times higher) (Fig 8A, 8C, 8I). Likewise, gill tissues from SML had higher CAT expression than laboratory samples (field OD: 0.93 ± 0.15, LDS OD: 0.69 ± 0.19; about 1.3-fold higher) (Fig 8B, 8D, 8I). Similar patterns appeared in digestive glands: field samples from SPI (mean OD 0.89 ± 0.12) and SML (mean OD 1.14 ± 0.17) had higher CAT expression than laboratory samples (SPI- 0.82 ± 0.19, SML- 0.67 ± 0.22) with about 1.1- and 1.7-time differences, respectively (Fig 8E-8H, 8J). Negative control tissues showed no IS signals (Fig 8K, 8L).

**Fig 8:**
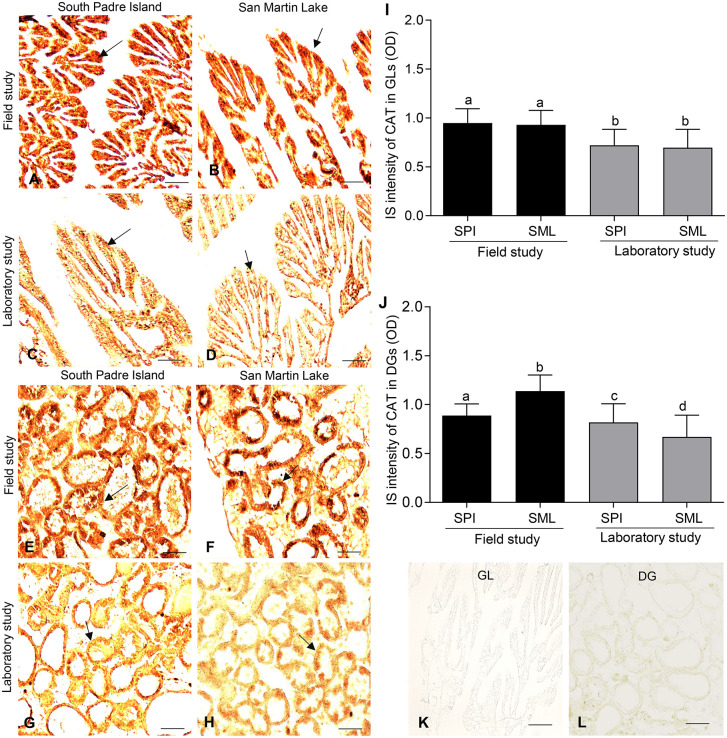
Detection of catalase (CAT) in tissues of wild-caught oysters collected for field and laboratory studies. Arrows indicate CAT expression in the gills (A-D) and digestive glands (EH) of oysters collected from South Padre Island (SPI) and San Martin Lake (SML) in the Texas Gulf Coast. (I, J) Immunostaining (IS) intensity of CAT in the gills (GLs) and digestive gland (DGs). Each value represents the mean ± SD (N = 200-214). Different letters indicate significant differences (Kruskal-Wallis test with Dunn’s multiple comparison test, *P* < 0.05). (K, L) Negative control of CAT expression in the gill and digestive gland of oysters. OD, optical density. Scale bar = 100 µm.

## 4. Discussion

Bivalves serve vital ecological functions through their filter-feeding behavior, but this also makes them vulnerable to microplastic pollution [88]. Understanding how microplastics affect their physiology is crucial for assessing water and oyster health. Although numerous studies examine oyster physiology and microplastics, few include both field and laboratory depuration experiments. In this study, we explored the impacts of microplastics on oysters’ gills, digestive glands, and extrapallial fluid, focusing on size, mucus secretion, nitrative stress, and antioxidant responses. We also compared these findings with a study of depuration to evaluate their capacity to recover in clean environments and what that might mean for future conservation. Continued research is essential to fully understand the effects of microplastics on marine life.

Biochemical techniques, such as Nile Red staining, are essential for detecting microplastics in tissues [89]. This method helps isolate microplastics and distinguish them from other materials [90]. Since Nile red also stains lipids [91], we avoided shapes that lacked solid edges or showed intense staining when measuring microplastics in oyster gills and digestive glands. We hypothesized that the gills and digestive glands of oysters collected from SPI and SML would contain more and larger microplastics than those of oysters in our laboratory depuration study, because the laboratory oysters had been in a clean, stress-free environment for two weeks. Our results confirmed that field oysters indeed had larger microplastics, while laboratory oysters had smaller particles in both their gills and digestive glands. Two explanations could account for this: the aquarium oysters filtered only clean water, allowing microplastics to be flushed out; or larger microplastics were broken down into smaller fragments, resulting in a similar total amount of microplastics but at smaller sizes.

Notably, while few studies directly compare field and laboratory results, many researchers have employed Nile red for microplastic identification [82]. Dowarah et al. [84] studied microplastics in two bivalve species, Asian green mussel (*Perna viridis*) and saltwater clam (*Meretrix meretrix*), collected from three estuaries in India, and examined the implications for local communities consuming these bivalves. Using Nile red, they counted microplastics in each bivalve and per gram of soft tissue, providing data on microplastic ingestion among local populations. Similarly, de Guzman et al. [83] studied Manila clams (*Ruditapes philippinarum*) from aquaculture farms in Mokpo, South Korea, to assess microplastics consumption. The clams were categorized into small (SC) and large (LC), revealing a wide range of microplastic sizes and a significant difference in concentration, LC had more microplastics than SC. These findings highlighted the importance of Nile red staining for detecting microplastics, making it a valuable tool for future research. The results from our study using Nile red suggest that oysters in the Texas Gulf Coast are filled with microplastics, and that the amount in their tissues could help estimate human intake, encouraging oyster farmers and the food industry to learn more.

Sadly, pollution from human activity has severely impacted the aquatic environment. As a result, many organisms face various stressors and suffer severe consequences, including oxidative and nitrative stress [92]. In this study, we used immunohistochemical analysis to assess NTP expression in the gills and digestive glands of oysters, as NTP is a biomarker of nitrative stress [93]. Our results showed higher NTP expression in field oysters from SPI and SML, supporting our hypothesis that wild oysters face more stress from microplastics and contaminants. In contrast, laboratory oysters in calmer, cleaner conditions had lower NTP expression in tissues. Many studies have examined NTP expression in aquatic organisms, but few have focused on oysters and microplastics. One study by Ahmed and Rahman [94] examined three oyster groups exposed to different pesticide doses and assessed the effects on NTP expression. They had a control group, a low-dose group (0.5 ug/L Roundup, 0.5 ug/L 2,4-D, and 0.4 ug/L atrazine), and a high-dose group (1 ug/L Roundup, 1 ug/L 2,4-D, and 0.8 ug/L atrazine). The control oysters were acclimatized for five days without pesticide exposure. Their results showed that oysters exposed to higher pesticide doses had experienced increased NTP expression. Similarly, Nash and Rahman [95] studied how rising ocean temperatures affected Eastern oysters by keeping them at 24°C (control), 28°C (medium), and 32°C (high) for a week. They found that NTP expression increased in oyster tissues at 28°C and 32°C, indicating more stress. Recently, Atamanalp et al. [42] and Espina et al. [43] demonstrated that dietary administration of PE microplastics depresses immunity, alters tissue architecture, and induces oxidative stress in rainbow trout (*Oncorhynchus mykiss*) and European sea bass (*Dicentrarchus labrax*), respectively. Collectively, these results support the idea that microplastic pollution can directly increase both oxidative and nitrative stress in fish and shellfish species. Moreover, our findings shed light on stress levels in oysters from microplastic contamination of seawater and show that their stress decreases in clean, filtered water, leading to lower NTP expression (i.e., protein nitration) in tissues, which may lead to normal physiological functions.

Understanding mucus secretion in oyster tissues is particularly helpful for assessing their stress levels, as they use mucus to trap particles and protect their gills [96,97]. Oysters pump water, and mucus captures particles that are then transported to their gills. The food particles are encapsulated by mucus, allowing safe passage through the digestive system [96,98]. Notably, PAS staining is a technique used to detect mucus secretion in tissues [99]. Our study showed that microplastics affect mucus secretion in oyster gills and digestive glands, as revealed by PAS staining. Results indicated that gill samples from field oysters produced less mucus in both SPI and SML than laboratory samples. Although digestive gland samples from field oysters also produced less mucus than laboratory samples, the difference was more noticeable than in the gills. We could not identify a specific reason for this. These findings support our hypothesis that stress in field oysters reduces mucus production, whereas relaxed laboratory oysters produce normal amounts of mucus. Although few studies compare mucus production between field and laboratory oysters, some research shows how PAS can reveal mucin production in various tissues. A study by Meyerholz et al. [100] demonstrated how they used the PAS protocol to detect mucin, a glycoprotein and a major component of mucus, in both normal (“control”) and glycogen-depleted tissues. Their results showed higher mucin production in normal tissues than in depleted ones. The glycogen-depleted oysters were experiencing environmental stress, similar to our field oysters, and therefore secreted less mucus. In contrast, our laboratory oysters, like the control group in the study by Meyerholz et al. [100], were in less stressful conditions. These results suggest that oysters produce less mucus under stress, which may affect their feeding and digestion. They also show that mucus production can increase in clean, safe environments, inspiring action to protect the oceans. Conversely, Zhang et al. [101] studied PE particles in the intestines, hepatopancreases, and gills of crayfish (*Procambarus clarkii*) after 21 days of exposure. They used PAS staining to measure mucus secretion and found increased levels in the intestine. The crayfish were divided into four groups: a control group with no microplastics, and three experimental groups with 0.5, 1, and 2‰ microplastics added to their food. The results showed up to a 5-fold increase in mucus secretion with 2‰ microplastics. In this study, higher stress was linked to increased mucus secretion, whereas in ours, more stress was linked to less mucus. This result highlights the need for further research into the effects of microplastics on mucus production in aquatic organisms.

Extrapallial (EP) fluid, or body fluid, serves many functions for oysters [102]. We measured pH and glucose concentrations in EP fluid collected from oysters, providing insights into how microplastic pollution affects these parameters. EP fluid is located between the inner shell surface and the mantle [103]. This fluid plays several roles, including shell formation and maintenance, and the regulation of opening and closing behaviors [104,105]. Shells protect oysters’ soft tissue from danger, maintaining a strong, healthy shell is essential for their survival. EP fluid also supports immune responses, helping to defend against pathogens [106]. Our EP fluid pH results showed no significant difference between field and laboratory oysters. The EF fluid pH levels were similar, with only a slight increase in laboratory oysters. This result suggests that field oysters, which experienced greater stress, had a slightly more acidic pH than laboratory oysters. A study by Billah and Rahman [107] showed how increasing temperatures affect *E. coli* levels in relation to oyster pH. They had a control group and two warmer groups at 28°C and 32°C. Their results indicated that EP fluid pH decreased with increasing temperature, with the control group being the least acidic and the 32°C group the most acidic. Similar to our findings, higher temperatures or, in our case, greater microplastic exposure led to greater stress and lower pH levels.

Glucose is the primary energy source for bivalves, making it a key carbohydrate for measuring energy response to stress and a reliable biomarker for oyster health [108–110]. Unlike our pH results, EP fluid glucose levels decreased. Field oysters had higher glucose levels, though the difference was not statistically significant, in contrast to the slight decline observed in laboratory oysters. Billah and Rahman [107] also found that glucose levels increased with rising temperatures. Oysters exposed to the highest temperatures, presumably the most stressed, had the highest glucose levels. These results suggest that glucose levels tend to rise under stressful conditions, affecting growth and physiological functions.

Antioxidants, such as SOD and CAT, help reduce oxidative and nitrative stress in aquatic animals [111,112]. Under extreme stress, antioxidant enzymes can respond in two ways: either they work overtime to combat stress and restore balance, or the stress overwhelms them, causing their activity to decline and stress levels to rise [113,114]. Our study showed how microplastics influenced SOD and CAT expressions in oyster tissues under different stress levels. Field oysters had higher SOD and CAT expressions in the gills and digestive glands at SPI and SML. Interestingly, the intensity of SOD and CAT responses matched NTP expression levels, confirming their role in the stress response. Although NTP expression was high in field oysters, it was not so extreme that SOD and CAT were overwhelmed. These results suggest that higher stress levels correlate with increased antioxidant enzyme levels. Since laboratory oysters experienced less stress, their antioxidant expression was lower. Recently, Tlili et al. [115] showed that microplastic mixture (0.24 g of PE/PP mixture, ratio 1:1) exposure (4–7 days) at environmentally relevant conditions induced oxidative stress and CAT activity in the gills and digestive glands of the wedge clam (*Donax trunculus*). Moreover, Kong et al. [116] demonstrated that short-term exposure (7-day) to PE nanoplastics (80 nm, 1 mg/L) and 3,3’,4,4’-tetrachlorobiphenyl (PCB77, 0.1 mg/L) induces oxidative stress, increases both SOD and CAT activities, and causes histopathological damage in the gills, digestive glands, and intestine in white hard clam (*Meretrix lyrata*). Our results align with these findings, indicating that increased stress from microplastics or other environmental contaminants leads to higher antioxidant expression, unless the stress becomes too severe, in which case the antioxidants become ineffective. Moreover, our study, together with other findings, provides novel insights into microplastic toxicity to marine organisms and the underlying molecular mechanisms of their ecotoxicological effects.

## 5. Limitations

No matter how many advances are made, limitations always remain. First, we were limited by not testing microplastics in the sediments and water during collection, which would have provided more environmental context and strengthened our argument for cleaner oceans. Our second limitation concerns the other pollutants in the aquatic environment besides microplastics. While we focused on the effects of microplastics, it is important to recognize that other environmental contaminants, such as pesticides, pharmaceutical drugs, and heavy metals, may also have contributed to increased nitrative stress, reduced antioxidant activity, and lowered mucus production in oysters. Nonetheless, all these pollutants pose serious threats to marine life and can serve as indicators of water pollution. Additionally, we had difficulty determining the correct amount of dye to use during counterstaining and often, tissues appeared all pink, indicating insufficient counterstaining (methyl green). Additional staining would have strengthened our results by providing more comparable replicates. Finally, quantitative analysis could not be performed because PE fibers, especially the smaller or thinner ones, may have been underestimated. Despite these limitations, our study provides valuable information on microplastic pollution in wild-caught oysters along the Texas Gulf Coast and advances our understanding of the mechanisms underlying microplastic accumulation and persistence in oyster tissues.

## 6. Conclusion

Our study revealed multiple ways in which microplastics have impacted the tissues and body fluids of oysters found along the Texas Gulf Coast, and how they could return to healthier levels in a clean environment (i.e., a laboratory study). Our field study revealed that oysters with higher NTP expression, a stress biomarker, must cope with stress more intensively when ingesting microplastics, as they exhibited lower stress levels than those in laboratory conditions in clean aquariums. This finding demonstrates that oysters have a better chance in clean water and experience greater physiological comfort than in polluted water, where they face constant stress. Due to their high stress levels, field oysters also showed increased activity of antioxidant enzymes, SOD, and CAT. Given the essential role of mucus, we measured mucus production and found that field oysters produced less than laboratory oysters. This result indicates that under higher stress, their mucus production decreases, suggesting oysters in polluted waters struggle to maintain their physiological systems. These findings demonstrate that microplastic pollution affects marine and coastal environments by increasing nitrative stress and impairing the biological and physiological functions in marine bivalve mollusks. As plastic production continues to rise, stress levels in marine mollusks increase, disrupting their growth, reproduction, and development. The results of this study provide valuable insights into aquaculture systems, restaurants, fishermen, and stockholders regarding environmental pollution and microplastic contamination in oysters. Moreover, our findings suggest that oysters can recover and return to healthier conditions when placed in clean environments, highlighting the importance of microplastics pollution reduction efforts for the benefit of both marine ecosystems and organismal health.

## Abbreviations

ART-FTIR: attenuated total reflectance Fourier transform infrared
CAT: catalase
EP: extrapallial
IS: immunostaining
NTP: 3-nitrotyrosine protein
OD: optical density
PAS: periodic acid-Schiff
PCB: polychlorinated biphenyl
PE: polyethylele
PP: polypropylelen
ROUT: robust regression followed by outlier identification
SEM: scanning electron microscope
SOD: superoxide dismutase
SPI: South Padre Island
SML: San Martin Lake
